# The Old Man Must Go

**Published:** 1900-09

**Authors:** F. M. Davis

**Affiliations:** Houston, Texas


For the Texas Medical Journal.

### The Old Man Must Go.

F. M. DAVIS, M. D., HOUSTON, TEXAS.

### OUR DREAM.

In my imaginary perambulation, I met a squad of elderly looking men and in their front and rear were several officers; as the place where we met was shady, they came to a halt. One of them asked me if that was the road leading to the potter's field. My answer was in the affirmative. I then asked him to please tell me the nature of their visit to the potter's field. One said, "We are going there, never to return." Question, "What are your avocations?" "Why, some of us are physicians, some are ministers, and some are teachers." "Well, what profession do you belong to?" "Oh, I am a doctor." "Well then, as I am one myself, I feel that I can question you more freely. Now tell me what process of law seems proper to deal thusly with you?" "It is an organized force of recent date, founded upon the trend of public sentiment, and is now dignified by the term Forum." Well, that certainly is a new law to me." "Yes, but its principles have been culminating for the past twenty years, but have only recently taken shape and gone into force." "That name sounds like it might mean forefront. Does it mean to capture just those that have fallen behind in their professions?" "No, it could not mean only that, because I know that I have kept thoroughly posted all along the line of recent medical literature." "Yes, but have you embraced it as fast as it appeared?" "No, for in every instance I have stopped to weigh well my responsibility in the administration of medicine and more especially so in the whirl of surgical advancement, and although I am on my exit to oblivion, I am not ashamed to say so, neither do I regret my conservatism." "Would you object to giving me an instance or two of your conservatism where the Forum has condemned you." "No. In the treatment of appendicitis, I have in many instances trusted to medical treatment instead of the use of the knife." "Why did you decline to use the knife?" "Because these cases were in their incipiency, and I had no means of telling whether the caecum was involved or not, and if such had been the case then to have cut down and excised the appendix would not have relieved the patient, and the operation would have injured him." "What is principally your medical treatment in this disease?" "Laxatives, poultices, and rest in bed." "What per cent. of appendicitis, without the assistance of a physician, get well?" "If the nurse is intelligent, eighty per cent., and with skilled medical treatment without the knife, over ninety per cent." "Then how many are saved by resorting to the knife?" "I will refer you to the health reporters." "Well, how did you console yourself after you had been called to a case in its incipiency, and the symptoms under your treatment grew worse and when you operated you found possibly abscess of the peritoneum and perforation of the intestines?" "When I was called to a case in its incipiency, I had no fears of evil results, especially if the health of my patient was otherwise good. And truly, all the unfavorable cases that I have ever seen were those that had been neglected in the beginning, such as had refused to stop and recline, had been given no medicine to open up their bowels. This is the history of cases resulting in abscess and perforation of the bowels." "Well, are you opposed to operating in any instance?" "No. When the appendix and the approximised peritoneum are involved, without perforation of the caecum, nothing expedites recovery so greatly and so surely as a skillful operation. Yes, I have refused to operate in every instance where the case was mild. Therefore, I have been berated by the Young America in surgery and am now to be beheaded by the Forum. You see, my wrinkles, my ground-off molars and my discolored hirsute, with my conservatism in the use both of medicine and the knife, have sealed my fate. And another feature of my conservatism was in the fact that I did not pronounce every spasmodic attack of nympho-ovarialgia as growing tumor, which demanded the knife at ence. And neither did I in every slight tenderness over the region of the tubes say that it was salpingitis, or pyosalpingitis, and urge the necessity of extraction of the same." "Well would this not rapidly grow worse if let alone and possibly result in metritis, and may be endometritis?" "Well, those affections are supposed to be the cause of salpingitis and not the result." "What was your treatment in these cases?" "In the beginning, I generally used antaphrodisiacs, sedatives, poultices, and cathartics." "Now, doctor, is it not a fact that an old M. D. may read and even take post-graduate courses and there see the new systems demonstrated and explained, and when he returns home, in most instances, he will refuse to adopt them?" "Well, yes. Well, no. It is true his past success with many old usages is hard for him to forget and he only appears objectionally conservative because the trend of medicine today is heroic in the use both of the drug and the knife." "Now, doctor, what one thing would tend to raise the standard of medicine more than all things else?" "An inflexible law that no student could matriculate in any medical college until he can produce a diploma from an accredited literary college." "Ar'n't many of the best physicians that we have ever had in America literary undergraduates?" "Yes, but that is the only place where the line can be drawn." "What do you think will be the next step in the study of medical science?" "An honest effort of the medical man to gain control of his patient's mind, the art of which would prove a *sine-qua-non* in nerve hyperaesthesis."

"Well, old man, your molars are ground to the gum and your incisors have lost their enamel, the tread of the crow has left its imprint on your cheeks, and these are only in keeping with your sight. Therefore, I see there is no hope of staying the fiat of the Forum until you could join some social order and then select your own manner of suicide, but it is too late now. So, *good-bye, old man; you must go.*"

Now I approached another rather sad faced old gentleman like this. "Will you be kind enough to tell me what profession you belong to?" "Yes, I am a minister of the gospel." "Well, what grounds have the Forum for condemning you and sending you to the potter's field?" "Why, I have just gone a little over on the shady side of life, you see." "You look stout, quite young, and active. Now, is there not a sequel to this seeming rashness of the Forum?" "Well, possibly so, but where is the justice in such a movement?" "Yes, but you answer by asking me a question. Now

I ask: Is there nothing to give insight in the matter?" "Well, I will speak only from my own observation, that the church in spite of the ministry, has grown away from the plan of salvation as it is laid down in the New Testament." "What has been your teaching along that line?" "I preached Jesus Christ and Him crucified. I never failed to point out the terrors of eternal punishment, and while I directed them to a throne of grace, I taught there is a penalty for disobedience. I explained to them that God is love, and that they must serve him in order to be happy, and that they must be born again before they can enter the kingdom of heaven." "Well, what has the Forum to say against that doctrine?" "It says that this doctrine is non-progressive, too exacting, and too constricting: That the church must have a new Christ, a ministry of broader views; a more liberal gospel, a doctrine founded upon rationality and Divine tolerance. They must have word and pen pictures of the avenues and realms of eternal bliss in the richest and most glittering colors with no prospective failures in their arrival there, and that the starry crown is pledged to all." "Well, good-bye, old man, may the Lord be more merciful to you than men have been."

I turned half round and another man was looking me in the face. I questioned him thus: "My friend, what is your occupation?" "I am a teacher, sir." "Well, did you ever sing the diphthongs?" "No, I am not quite a hundred years behind the times, although I used to parse, and knew the ten parts of a speech in English grammar as well as an ordinary boy of seven today knows his alphabet." "Well, please, what is the trouble between you and the Forum?" "Oh the Forum just simply says I have to go." "Well, have you kept up with the radical changes that have been going on in the school-room for the past decade or so?" "Well, I have been all the while actively engaged in the construction of whatever changes have been made for the last twenty years." "Please mention some features of changes in school management." "Grading is one of the most eventful of the many changes of late. The care with which children are examined, selected and classified is regarded as a tenstrike in furthering them in their studies, and next, objective lessons and diagraming go straight to the conception of the child. Discipline and child-study have become most prominent in the line of teachers' duties." "What do you think of the probable abridgment of all English words that seemingly have a superfluous amount of letters, and the withdrawal of all silent letters?" "I am opposed to the change." "Why?" "Because it would destroy the key to the derivation of those words. You see the way words are spelled in most instances gives insight to their meaning; therefore the ordinary scholar hardly has to refer to his lexicon in his reading." "What do you regard as the highest attribute of a teacher?" "I believe the art and power of concentrating the minds of his pupils on their work is highest." "Now, can you lay aside your present grievance and give me a laudable reason for this wholesale displacement of elderly men and the universal installment of young men?" "Yes, it is due to the educational wave that struck this country fifteen or twenty years ago, which has resulted in there being among us today five hundred educated young men where thirty-five years ago there were not more than fifteen." "Is the classical young man of today upon *average* more thorough in his collegiate course just finished than the ordinary classical young man of thirty-five years ago?" "No, but he has the advantage of being five years younger." "How is that?" "Why, the present system of teaching expedites the student in conception to that extent. The teacher's manner of imparting knowledge, here of late, has become the wonder of the age. Therefore, the college young man today at twenty is equal to the college young man thirty-five years ago at twenty-five years of age." "Now, tell me why the educated man of thirty or thirty-five years ago, with all his subsequent experience, is no longer sought after." "It is due to the fact that he is not so sprightly and is less comely than in his earlier days. He is not so quick to recognize his old friends and cannot make as many new ones as when young." "Well, that argument means that experience nowadays goes for naught." "Yes, the world of business is moving too swiftly for any investigation as to qualifications." "What do you think will be the next step in the science of pedagogy?" "Mind study; the delineations of the cranium, its contour relative to human character. This will soon be incorporated in the curriculum of studies for the teacher, which will enable him to detect the line of study each child is best capacitated for." "Well, this interview has been very instructive to me, and while I doubt the fact of your having reached the zenith of your usefulness, and the world is evidently better by your having lived, yet your pillow has not been softened by the world, and you now have to bow to the fiat of the Forum. So, good-bye, old man; you must go."

Now, I started off at a rapid gait because I felt so alarmed at the future outlook. *Just then came my close call.* I was commanded to halt; at the same instance something brushed my hat and shoulder. I turned my head to the right and beheld a man dressed in blue, holding a long pole with a wire loop at its end. He had made a pass at my head. He asked me if I was not going the wrong way. I answered, "No, sir." He said, "I am what is called the pound man for the Forum, and you look a little like you might be getting on the shady side of life." I said, "No, no, I am only a little weather beaten, prematurely old, you see, just in looks, that's all, due to overwork and loss of sleep. I am a *doctor* and used to practice out west. The circumstances were these: I located in a large neighborhood, and at one side of that community lived a man by the name of Bill Cain, and at the other extreme lived a man by the name of Frank Harper. These two men had large families, and they had a great deal of sickness at all seasons of the year. They had a great deal of influence in the neighborhood, and while they never paid me one cent for any work that I ever did for them, yet during the ten years that I practiced there I never received three calls in succession that one was not either to Bill Cain's or to Frank Harper's. You see it would not have done to refuse them service, or to have incurred their displeasure in any way, because it would have thrown me out of business. I would have bankrupted without the benefit of an assignee. Therefore, these little imprints of the foot of the crow about my mouth and cheeks are all premature. They are just long drawn out care and anxiety from conscious responsibility while in charge of those influential families, Cain and Harper." "Well, you pretend that you are too young for the pound or potter's field. How about that hole in your arm. Is not that a war scar?" "No, no, don't take me to be old enough for that. You see, it was like this: I used to ride a mule, and he was one of the best saddle animals you ever saw. I called him Simeon. He had but one fault; that was, he would tip his toe occasionally, and when that occurred, he never recovered himself until he fell completely down and rolled over. So you see I was going at a rapid gait to Bill Cain's one night, when old Simeon tipped his toe, and down he came in a pile, and when he rolled over he caught my arm, and the cuff button was thrust into my wrist." "Yes, but how about that hole just opposite, on the other side?" "Well, as I was going to say; at another time, when I was going in a sweeping pace down grade at night to Frank Harper's, old Simeon again tipped his toe and down we came together, and when he rolled over, the cuff button happened to be on the other side and ran into my arm; wonderful coincidence, indeed." "Well, how about that white spot on your head? Did that bloody cuff button do that?" "No, that occurred like this: You see, we were going in a gallop at night to Bill Cain's, when Simeon ran under a mesquite limb and just literally scalped me, and when the skin grew back, the hair came out white and has been that way ever since." "Yes, but your jaw teeth are a little worn." "Well, that was done in Calhoun county, Miss. When I was a boy I used to try to crack hickory nuts with my teeth, and just drove them up into my jaws." "Well you argue so well that I, as an agent of the Forum, will let you go this time, but you look to be anywhere from forty to a hundred years old."

As I left, I muttered to myself, like this: "Well, old man, I don't know how to tell you good-bye, but it looks a little like you too will soon have to go."

